# The Role of Non-coding RNAs in Diabetic Nephropathy-Related Oxidative Stress

**DOI:** 10.3389/fmed.2021.626423

**Published:** 2021-04-20

**Authors:** Xiaoyun He, Gaoyan Kuang, Yi Zuo, Shuangxi Li, Suxian Zhou, Chunlin Ou

**Affiliations:** ^1^Department of Pathology, Xiangya Hospital, Central South University, Changsha, China; ^2^Department of Orthopedics, The First Affiliated Hospital of Hunan University of Chinese Medicine, Changsha, China; ^3^Department of Endocrinology, Affiliated Hospital of Guilin Medical University, Guilin, China; ^4^Department of Pathophysiology, Hunan University of Medicine, Huaihua, China; ^5^National Clinical Research Center for Geriatric Disorders, Xiangya Hospital, Central South University, Changsha, China

**Keywords:** diabetic nephropathy, oxidative stress, ncRNA, mircoRNA, therapeutic target

## Abstract

Diabetic nephropathy (DN) is one of the main complications of diabetes and the main cause of diabetic end-stage renal disease, which is often fatal. DN is usually characterized by progressive renal interstitial fibrosis, which is closely related to the excessive accumulation of extracellular matrix and oxidative stress. Non-coding RNAs (ncRNAs) are RNA molecules expressed in eukaryotic cells that are not translated into proteins. They are widely involved in the regulation of biological processes, such as, chromatin remodeling, transcription, post-transcriptional modification, and signal transduction. Recent studies have shown that ncRNAs play an important role in the occurrence and development of DN and participate in the regulation of oxidative stress in DN. This review clarifies the functions and mechanisms of ncRNAs in DN-related oxidative stress, providing valuable insights into the prevention, early diagnosis, and molecular therapeutic targets of DN.

## Introduction

Diabetic nephropathy (DN) is one of the main complications of diabetes mellitus (DM) and the main cause of diabetic end-stage renal disease (ESRD), resulting in the disability and death of patients with DM (1, 2). DN is characterized by progressive renal interstitial fibrosis, which is accompanied by a series of pathological changes, including excessive accumulation of extracellular matrix (ECM) components, thickening of the glomerulus and tubular basement membrane, and increased formation of glomerular and tubular basement membrane matrix (3, 4). The increased prevalence of DM has led to an increase in the incidence of DN. DN is a leading cause of ESRD (5, 6), which is one of the major health problems worldwide.

The imbalance between oxidants and antioxidants is called oxidative stress (OS) and occurs when the body is subjected to various harmful stimuli, leading to the injury of tissue and cells (7). OS exists in all stages of DM development, and hyperglycemia is the main factor promoting OS (8–12). In addition, the advanced glycation end products (AGEs) associated with hyperglycemia (13), reactive oxygen species (ROS) (14, 15), the protein kinase C (PKC) pathway (16), and the renin-angiotensin system promote the occurrence of OS (17) and its maintenance, and cause the development of DN (8). Furthermore, the abundance of mitochondria in kidney tissue renders it more vulnerable to OS (18). Intrarenal OS, which also plays a vital role in the pathogenesis of DN, causes chronic inflammation of the kidney, and glomeruli and tubular hypertrophy (19).

Ribonucleic acid (RNA) is divided into two categories according to its characteristics: coding RNA and non-coding RNA (ncRNA). Research on ncRNAs has demonstrated that these molecules are not simply “junk” transcription products but functional regulatory molecules that mediate cellular processes. Many ncRNAs affect specific cellular biological responses, and are key regulatory molecules in the course of disease (20). For example, small ncRNAs, such as, microRNAs (miRNAs), may act as proto-oncogenes or tumor suppressor genes in cancers (21, 22); circular RNAs (circRNAs) participate in the development of tumors, neurological diseases, and rheumatic diseases (23–25); small nucleolar RNAs (snoRNA) participate in tumors, metabolic stress, and other diseases through modification (26, 27); long-chain ncRNAs (lncRNAs) have been linked to cancer, diabetes, heart failure, hypertension, kidney disease, and other diseases (28–32). Thus, ncRNAs are a new hot spot in epigenetic research. Although, the role of several ncRNAs in disease pathogenesis has been revealed, their specific regulatory networks have yet to be studied.

## Classification of ncRNAs

The Human Genome Project led researchers to discover that protein-coding sequences only account for nearly 2% of the human genome, while the remaining non-coding regions were considered “junk areas.” The development of computational biology and the popularization of genome sequencing technology showed that these “junk regions” are transcribed into large amounts of RNA, of which nearly 74% are ncRNAs (33, 34). According to their functions and sizes, ncRNAs are roughly divided into three categories: housekeeping ncRNAs, small RNAs (sRNAs), and lncRNAs; circRNAs constitute a special type of lncRNA. Housekeeping ncRNAs are essential for cellular activities, and include ribosomal RNAs (rRNAs), transfer RNAs (tRNAs), small nuclear RNAs (snRNAs), and snoRNAs (35). sRNAs generally refer to ncRNAs shorter than 200 nucleotides (nt). According to their species origin, they are divided into two categories: bacterial sRNAs and eukaryotic sRNAs. Eukaryotic sRNAs include miRNAs, small interfering RNAs (siRNAs), and PIWI-interacting RNAs (piRNAs). lncRNAs have lengths of more than 200 nt (36), and are widely transcribed in the genome. According to their transcriptional location, molecular characteristics, or position relative to mRNAs, lncRNAs are divided into long intervening/intergenic ncRNAs, which are located in the gap between two mRNAs and have an independent transcription; natural antisense transcripts, which reversely overlap with an mRNA exon; promoter upstream transcripts (prompts); enhancer RNAs, which are transcribed from enhancers of protein-coding genes; and circRNAs (37, 38).

## Biological Characteristics of ncRNAs

**Figure 1:**
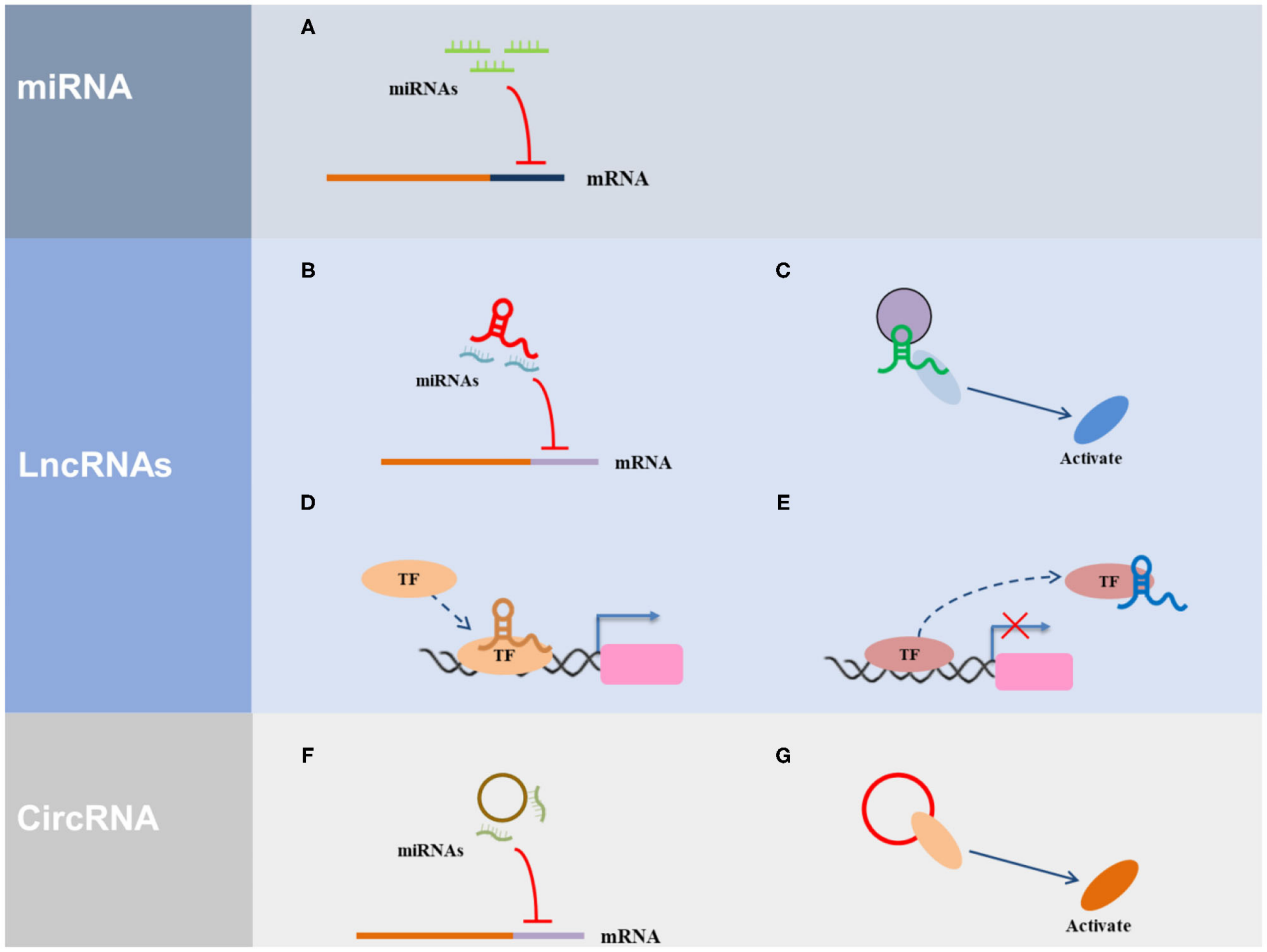
The regulatory mechanism of ncRNAs. **(A)** miRNA can direct bind with the 3′ UTR of the target gene. **(B)** LncRNA serves as ceRNA to interact with miRNAs. **(C)** LncRNA acts as a scaffold *via* recruiting and interacting with proteins and regulate the activity of proteins. **(D)** LncRNA acts as a guide to promote the gene expression *via* recruiting transcription factors (TF) to the region of the gene promoter. **(E)** LncRNA acts as a decoy *via* interacting with TF to inhibit transcriptional regulation. **(F)** CircRNA serves as ceRNA to interact with miRNAs. **(G)** CircRNA acts as a scaffold *via* recruiting and interacting with proteins and regulate the activity of proteins.

ncRNAs are widely involved in important biological functions, such as, the development and differentiation of cell development and differentiation, reproduction, cell apoptosis, and cell reprogramming, and are closely associated with disease development and progression. To date, many studies on miRNAs, lncRNAs, and circRNAs have been conducted (Figure 1).

### miRNA Function

It is generally believed that miRNAs bind to RNA-induced silencing complexes in the cytoplasm, recognizing, and binding their matching target sequence (usually in the 3′ untranslated region of the protein-coding gene) in a sequence-specific manner, thus, regulating the degradation of target gene mRNA and/or its translation to inhibit gene expression. However, some studies have suggested that some miRNAs, such as miR-320, miR-373, miR-122, and miR-483, may also promote or inhibit the expression of target genes in the nucleus (39). Nuclear miRNAs may recognize and bind target sequences in gene promoters and other DNA regulatory elements in a sequence-specific manner, and then recruit proteins or complexes responsible for epigenetic modification, which results in chromatin remodeling, resulting in transcriptional activation or gene silencing. Alternatively, nuclear miRNAs inhibit lncRNAs near target genes, thereby regulating gene expression (40). At present, the reasons, mechanisms, and functions of miRNA accumulation in the nucleus remain unclear and require further investigation. Based on the role of miRNAs in regulating target gene expression, the functions of miRNAs are determined by the functions of their target genes. If the target genes regulate cell proliferation, apoptosis, differentiation, and other important biological functions, the miRNAs will play an important role in these biological functions. miRNAs have obvious cell line specificity in the regulation of target genes. Different miRNAs in the same cell line have different roles, and the same miRNAs in different cell lines also have different functions. Hundreds of miRNAs in cells have a complex regulatory role in tens of thousands of protein-coding genes, forming a genome-wide expression regulatory network that keeps protein expression at normal levels. Hence, miRNA abnormality often affects important physiological processes and triggers the occurrence of major diseases. Studies have shown that the abnormal regulation of miRNAs is a common feature of many diseases (41), making them a novel interventional target. Due to the stability of miRNAs in organisms, their application in the diagnosis and prognosis of disease will be useful. In short, miRNAs have tremendous potential in disease treatment and translational medicine.

### lncRNA Function

lncRNAs are linear ncRNA transcripts with complex structures, and are widely expressed in mammalian genomes. Compared with mRNA, lncRNAs have low expression levels, high tissue specificity, and strong temporal and spatial expression specificity (42). It is estimated that more than 10,000 lncRNAs exist in the human genome. However, biochemical identification and functional research is still in its infancy. At present, the functions of only ~100 lncRNAs are understood. These lncRNAs regulate the expression or activity of target genes at the DNA, RNA, and protein levels, and are widely involved in a variety of important regulatory processes, such as, the inactivation of the X chromosome, genomic imprinting, stem cell pluripotency, somatic cell reprogramming, chromatin remodeling, the formation of nuclear substructures, and intranuclear transport, and are associated with the occurrence and development of many human diseases, such as, tumors, cardiovascular diseases, and neurological diseases (43–47). lncRNA regulation of target protein-coding genes occurs both during and after transcription, as lncRNAs may be located in the nucleus or cytoplasm. It has been shown that 30% of all lncRNAs are located in the nucleus, 15% in the cytoplasm, and the rest are found in both the nucleus and the cytoplasm.

Recently, with increasing research and the development of sequencing technology, an increasing number of lncRNAs and functions have been reported. Currently, there are over 20,000 lncRNA annotations, which is more than protein-coding gene annotations. For the whole genome of a cell, the expression of lncRNAs is much lower than that of protein-coding genes, and exhibits obvious tissue and cell line specificity. Some lncRNAs begin to function at certain stages of eukaryotic development. Because lncRNAs regulate the expression of protein-coding genes in different ways, lncRNAs are involved in many important biological processes, including heredity, development, cell cycle, and changes in chromosome structure. lncRNA abnormalities are involved in the development of many common diseases (45, 47, 48).

### circRNA Function

circRNAs are special ncRNAs that form closed loop structures through covalent bonds (49, 50). circRNAs are believed to mainly exist in the cytoplasm, with a small amount found in the nucleus (51, 52). circRNA expression is extremely abundant in eukaryotes and is evolutionarily conserved. Although, they do not encode proteins, they interact with proteins, regulate the variable splicing process of pre-mRNA, and regulate the maturation of rRNAs (53, 54). In addition, circRNAs play an indispensable role in the normal physiological processes of biological reproduction, growth, and aging, and are involved in the occurrence and development of neurological diseases, autoimmune diseases, cardiovascular diseases, and tumors (23, 55). Some circRNAs regulate the expression of protein-coding genes by competitively binding miRNAs (56). Furthermore, both lncRNAs and circRNAs are used as competitive endogenous RNAs to combine with miRNAs, forming an interactive regulatory network (57). The development of bioinformatics technology will allow further understanding of the functional role of these three types of ncRNAs, which will help us clarify the pathogenesis of certain diseases and develop therapeutic strategies and drugs.

### Other ncRNAs Function

Other ncRNAs mainly contain snoRNAs, which can be divided into three categories based on their structural elements: box C/D snoRNA, box H/ACA snoRNA, and MRP RNA (58, 59). The main snoRNAs in cells are box C/D and box H/ACA snoRNAs. In addition, snoRNAs can be classified based on their gene organization, into independently coding snoRNA and intron coding snoRNA. snoRNAs can be classified based on their gene organization, ranging from independently transcribed genes under the control of independent promoters, to intronic coding units, which lack an independent promoter and are encoded in introns of protein-coding host genes. snoRNA participates in the biosynthesis of eukaryotic ribosomes, mainly to guide the modification of nucleotides at specific sites and participate in rRNA shearing (59, 60). Cajal body-specific small RNA (scaRNA) is similar to snoRNA and has box C/D or boxH/ACA structural elements (61). scaRNA guides the nucleotide modification of snRNA. Moreover, a special class of molecules including U85, U87, U88, U89, with both box C/D, and box H/ACA domains were found, which can simultaneously guide the ribose methylation and pseudouracilization of U5 and U4 snRNA. Vertebrate telomerase is a box H/ACA telomerase box, which may be essential for *in vivo* telomerase accumulation, 3′ end processing of the telomerase RNA precursor to ensure the stability of mature RNA, and telomerase activity. Mutations in human telomerase box H/ACA motif or the bound small nucleolar ribonucleoprotein (snoRNP) dyskerin cause multi-system genetic diseases (62). Although, some small RNAs have the typical structure of box C/D or box H/ACA, and primarily guide the chemical modification of other RNAs, including rRNAs, tRNAs, and snRNAs, a large subclass of snoRNAs called orphan snoRNAs cannot find complementary sequences that match rRNA or snRNA (63). It has been reported that many RNAs that are not directly related to ribosomal biosynthesis, including a small number of mRNAs, can temporarily stay in the nucleolus. Moreover, in yeast, the RNAse P-mediated 5′ end processing of some tRNA precursors occurs in the nucleolus. Therefore, it is possible that orphan snoRNAs act on RNAs other than rRNA and snRNA.

## DN Pathogenesis

DN is characterized by pathological albumin excretion or albumin/creatinine ratio in the urine of patients with diabetes and a decrease in the glomerular filtration rate (64–66). Pathological changes in DN, such as, glomerulus enlargement, basement membrane thickening, and accumulation of glomerular mesangial and tubular ECM, lead to glomerular and tubular interstitial fibrosis, and even hardening (67, 68). DN is the main cause of ESRD worldwide and is related to the incidence and mortality of cardiovascular events (8, 69, 70). Risk factors for the occurrence and development of DN include increased inflammation, oxidation markers, AGEs, ROS, elevated levels of transforming growth factor-β (TGF-β), elevated PKC levels, abnormal polyol metabolism, uric acid levels, a long history of DM, age at diagnosis, race, systemic or glomerular hypertension, proteinuria, genetic susceptibility, insulin resistance, and diet composition (69, 71).

**Figure 2:**
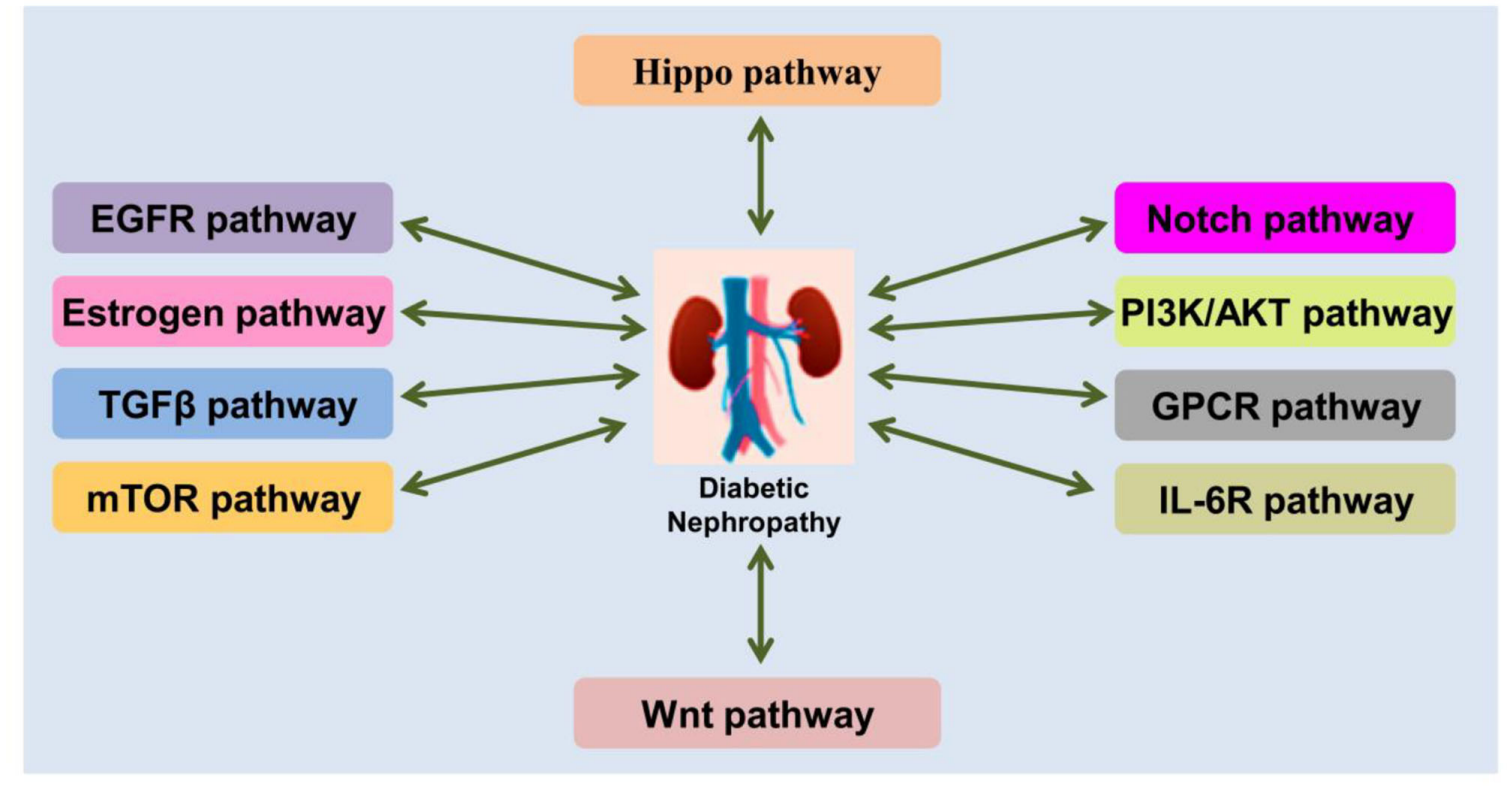
Schematic demonstration of the crosstalks between DN and signaling pathways.

The pathogenesis of DN is complex process, involving in a series of signaling pathway changed (Figure 2). Moreover, the pathogenesis of DN depends on the following aspects: (1) Genetic susceptibility factors. Specific single nucleic acid polymorphisms in susceptibility genes have been associated with DN, and therefore, research on this area is helpful for the prevention of DN (72–74); (2) Abnormal glucose metabolism. Hyperglycemia promotes the occurrence of a variety of pathophysiological processes, including the activation of the polyol pathway (75, 76) and the generation and accumulation of AGEs (77–79); (3) Inflammatory reactions. DN is an inflammatory disease caused by a metabolic disorder As such, inflammatory reactions accompany the entire process of DN development, which result in the gradual scarring of the renal glomeruli, known as glomerulosclerosis (67, 80, 81); (4) Cytokines. Proinflammatory cytokines affect hemodynamics, promote cell proliferation, and increase ECM secretion and renal interstitial fibrosis, thus participating in the occurrence and development of DN (82); (5) OS. Excessive ROS production in the body activates PKC and the polyol pathway, leading to an increase in AGEs, and cytokine release, eventually resulting in severe pathological changes in the kidneys and promoting the occurrence and development of DN (83); (6) Endoplasmic reticulum stress (ERS). Excessive ERS may cause OS by promoting an increase in ROS, thereby damaging the kidneys (83–85) and participating in the development of DN; (7) Autophagy. This is the process whereby damaged proteins and organelles are decomposed after a stress response, and this plays an important role in maintaining cell homeostasis (86). The activation of the mechanistic target of rapamycin (mTOR) (87, 88) and the reduction of 5′ AMP-activated protein kinase (89) and Sirtuin 1 (SIRT1) (90) attenuate autophagy-related activities, and this attenuation has been linked to the pathogenesis of DN; (8) Exosomes and extracellular vesicles. Recently discovered, exosomes and extracellular vesicles are closely related to the occurrence and development of DN. The expression of exosomes is abnormal in DN, and the DNA, RNA, and protein contained in them are involved in the pathogenesis of DN and are used as molecular markers of DN (91, 92). In general, the pathogenesis of DN is complex, and what is currently known may just be the tip of the iceberg. Therefore, the integration of multiple aspects of the disease is crucial for the development of effective treatments.

Previous studies have reported that oxidative stress plays an important role in the occurrence and development of DN (3, 93–95). Hyperglycemia induces renal cells to produce large amounts of ROS, which increases oxidative stress. During oxidative stress, multiple signal pathways such as, glucose oxidation, production of advanced glycation end products (AGE), activation of protein kinase, hexosamine, and polyol pathways are involved in the metabolic regulation of glucose and lipids (96). Oxidative stress in the kidneys usually results in the massive production of ROS by peroxidase, which induces renal fibrosis and inflammation, and leads to tissue injury by promoting lipid peroxidation, DNA damage, and mitochondrial dysfunction (97). Under normal physiological conditions, ROS play a significant role in the regulation of cell proliferation, differentiation, apoptosis, and immune defense, while under the pathological conditions of diabetes, excessive ROS production in the kidneys stimulates the recruitment of inflammatory cells and the release of large amounts of inflammatory factors, growth factors, and transcription factors (71), thereby altering kidney structure and function, and promoting DN (98). Oxidative stress also accelerates the occurrence and development of DN by damaging the podocytes in the glomerular filtration barrier. The mechanisms whereby oxidative stress induces podocyte injury include ROS-induced mitochondrial dysfunction, activation of the mitogen-activated protein kinase (MAPK), and NF-κB signaling cascade, and oxidative damage of DNA (99). In addition, oxidative stress contributes to the development of glomerulosclerosis. ROS activate signaling pathways such as angiotensin II/transforming growth factor β1 (TGF-β1)/smad, protein kinase C (PKC), and NF-κB, inducing the deposition of extracellular matrix. However, signaling factors such as angiotensin II, TGF-β1, and PKC also facilitate the generation of ROS, aggravating DN and oxidative stress damage. Moreover, oxidative stress is involved in the development of renal tubular fibrosis. ROS stimulate the expression of a variety of pro-fibrotic growth factors such as TGF-β1, vascular endothelial growth factor (VEGF), and connective tissue growth factor, further boosting the deposition of extracellular matrix proteins and renal function damage (100). Therefore, it is extremely crucial to explore the mechanisms whereby oxidative stress participates in the occurrence of DN.

## Relationship Between ncRNAs and Diabetic Nephropathy-Related Os

**Figure 3:**
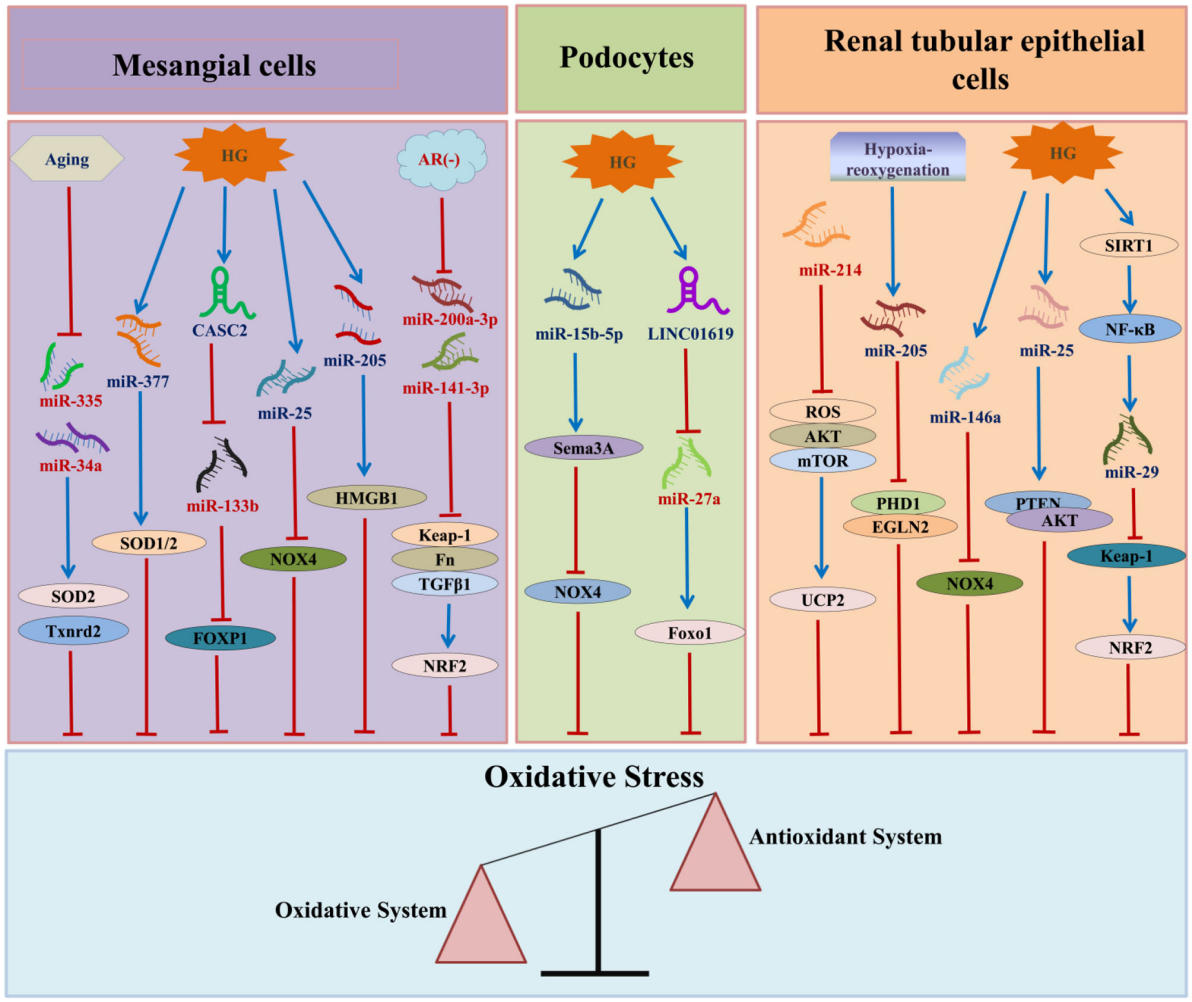
The relationship between ncRNAs and DN-associated oxidative stress. HG, high glucose; AR, aldose reductase; CASC2, cancer susceptibility candidate 2; HMGB1, high mobility group box 1; SOD, superoxide dismutase; NOX4, nicotinamide adenine dinucleotide phosphate oxidase 4; FN, fibronectin; TGF-β1:transforming growth factor β1:TXNRD2, thioredoxin reductase 2; FOXP1, forkhead box P1; NRF2, nuclear factor erythroid E2-related factor 2; SEMA3A, semaphorin 3A; Foxo1, Forkhead box 1; ROS, reactive oxygen species; AKT, protein kinase B; mTOR, mechanistic target of rapamycin; UCP2, uncoupling protein 2; SIRT1, Sirtuin 1; NF-κB, nuclear factor-kappa B; PTEN, phosphatase and tensin homolog; PHD1, prolyl hydroxylase 1.

In recent years, various studies have confirmed that ncRNAs participate in the occurrence and development of DN by regulating OS. Among the ncRNAs that regulate OS in DN, miRNAs are the most widely studied, followed by lncRNAs and circRNAs (Figure 3).

### miRNAs and Diabetic Nephropathy-Related OS

miRNAs are post-transcriptional regulatory RNAs with a length of 18–23 nt that are widely present in eukaryotes, do not encode proteins, and are able to inhibit gene expression through specific interactions with target genes. Altered miRNA expression levels may generate OS and ultimately result in the development of disease. The relationship between miRNA and OS in the pathogenesis of DN has also become a research hotspot in recent years.

#### Up-Regulated miRNAs

The miR-23a/27a/24-2 cluster upregulates c-jun N-terminal kinases (JNKs) to induce caspase-dependent and caspase-independent cell death in human embryonic kidney cells (HEKT293), which is accompanied by an increase in ROS (101). In addition, overexpression of the miR-23a/27a/24-2 cluster results in changes in HEKT293 ERS and mitochondrial membrane permeability (102), suggesting that the cluster is closely related to OS in the kidney. Uncoupling protein 2 (UCP2), a negative regulator of ROS generation (103), is the target gene of miR-24 in the kidney; The downregulation of hsa-miR-24-3p results in UCP2 upregulation and subsequent reduction in ROS production (104). Shao et al. (105) found that miRNA-3550 is upregulated in the kidneys of DN rats and is related to the Wnt/β-catenin signaling pathway. miR-27a directly targets nuclear factor erythroid 2-related factor 2 (NRF2), interfering with ROS homeostasis in DN, while adipokinin omentin 1 upregulates NRF2, and reduces OS by inhibiting miR-27a, restoring kidney function in type 2 diabetic db/db mice (106).

miRNA-452-5p expression is increased in high glucose (HG)-treated HK-2 cells (a renal tubular epithelial cell line), which is accompanied by an in increase in ROS and malondialdehyde (MDA) levels, and a decrease in SOD levels (107). However, these changes are reversed by interfering with miR-452-5p activity (108), which suggests that miR-452-5p is involved in the HG-induced OS response of renal tubular cells. Compared to controls, the levels of microRNA-377 have been shown to be consistently up-regulated in *in vitro* and mouse diabetic nephropathy models. The activity of miR-377 leads to reduced expression of p21-activated kinase and superoxide dismutase, which enhances fibronectin (FN) production. Thus, the overexpression of miR-377 in diabetic nephropathy indirectly leads to increased FN production. In addition, miR-377 targets SOD1 and SOD2 in human mesangial cells (HMCs) (109). Bioinformatics analysis and verification of the miRNA expression profiles in kidneys of young and old rats demonstrated that mitochondrial *Sod2* and thioredoxin reductase 2 (*Txnrd2*) are the targets of miR-335 and miR-34a, respectively. In aging MCs, miR-335 and miR-34a are significantly upregulated, while *Sod2* and *Txnrd2* are significantly downregulated, which is consistent with the production of ROS (110). Kato et al. (111) showed that TGF-β activates protein kinase B (AKT) in glomerular mesangial cells by inducing miR-216a and miR-217, leading to glomerular mesangial cell proliferation and hypertrophy. Zhang et al. (112) found that miR-133b is upregulated in the serum and HMCs of patients with DN, and that miR-133b targets forkhead box P1 (*FOXP1*). The upregulation of lncRNA CASC2 inhibits HG-induced HMC proliferation, ECM accumulation, and OS *via* the miR-133b/FOXP1 regulatory axis. Podocyte damage is a sign of DN, and is induced *via* ERS by the upregulation of miR-27a, which negatively targets forkhead box 1 (FOXO1) (113).

After analyzing miRNAs in the serum of healthy controls, patients with type 2 DM, and patients with DN, Regmi et al. (114) found that serum miR-99b and miR-122 levels are significantly increased in DM and DN group patients, while those of miR-20a, and miR-486 are decreased, and that the levels of these miRNAs are significantly related to albuminuria, glomerular filtration rate, blood sugar, and blood lipid levels. Moreover, target gene prediction of these four miRNAs revealed that they regulate OS, inflammation, and apoptosis (114). The emergence of RNAseq technology has facilitated the discovery of miRNAs related to OS in DN and the identification of their target genes, enabling in-depth research on the pathogenesis of DN.

#### Down-Regulated miRNAs

Renal tubular epithelial cells are one of the main cells that absorb glucose; however, long-term hyperglycemia directly causes their damage, and induces dysfunction through OS. In HG-treated HK-2 cells, the deacetylase activity of SIRT1 is weakened, resulting in a decrease in NF-κB activity. NF-κB regulates the expression of miR-29, which targets *KEAP1* by directly binding to its promoter. Downregulation of miR-29 in response to HG enhances the expression of KEAP1, and reduces NRF2 levels through ubiquitination, thereby inducing the damage of renal tubular epithelia (115). Moreover, miR-29 expression is negatively correlated with serum creatinine levels and creatinine clearance in diabetic rats (115). Yang et al. (116) found that miR-214 inhibits OS in DN and enhances the expression of UCP2 through the ROS/AKT/mTOR signaling pathway in HK-2 cells. The expression of UCP2 attenuates mitochondrial ROS activity, thereby exerting an antioxidant effect.

Superoxide derived from nicotinamide adenine dinucleotide phosphate oxidase (NOX) plays a key role in hyperglycemia-derived OS in DN; OS production by NOX mediates matrix accumulation and renal fibrosis in DN (117, 118). In HG-treated MCs and the kidneys of streptozotocin (STZ)-induced diabetic rats, the expression of miR-25 is significantly reduced, while the mRNA and protein levels of NOX4 are increased (119). MCs transfected with antagomiR-25 showed a considerable increase in the mRNA and protein levels of NOX4 (120). These results are consistent with the increased OS and diastolic dysfunction observed in the hearts of hypercholesterolemic rats when the expression of miR-25 is decreased (121). Thus, the miR-25-NOX4-OS axis seems to play a common role in kidney and heart diseases. In addition, miR-25 is downregulated in kidney biopsy tissue and serum of patients with DN, and is inversely proportional to proteinuria. Moreover, HG-treated HK-2 cells show decreased miR-25 levels in a time-dependent manner. Overexpression of miR-25 reduces the generation of ROS in HK-2 cells; the mechanism may be related to the activation of the phosphatase and tensin homolog/AKT pathway (122). Similarly, Wan et al. (123) confirmed that miR-146a expression is inhibited and NOX4 levels are increased in a DN mouse model. In addition, overexpression of miR-146a in HK-2 cells inhibits NOX4 expression, reducing ROS production, OS, and inflammation levels (123), which suggests that miR-146a has an anti-inflammatory and oxidation modulating effect in DN.

Wei et al. (124) reported that aldose reductase (AR) negatively regulates the expression of miR-200a-3p/miR-141-3p in MCs. In STZ-induced diabetic mice, AR deficiency significantly increases the miR-200a-3p/miR-141-3p levels in the renal cortex, which is accompanied by the significant downregulation of *Keap1, Tgf*β*1/2*, and *fn1*, and the prominent upregulation of *Nrf2*. Therefore, the inhibition of AR and the restoration of the miR-200a-3p/miR-141-3p levels may be a potential research direction for the treatment of DN.

The expression of miR-506-3p is downregulated in HG-treated HK-2 cells and the serum of patients with DN, while overexpression of miR-506-3p inhibits inflammation, OS, and pyroptosis in HG-treated HK-2 cells (125). miR-15b-5p is significantly decreased in HG-treated podocytes, which is accompanied by increased levels of podocyte apoptosis and OS (112). Overexpression of miR-15b-5p inhibits cell apoptosis, decreases the expression of the OS-related markers *MDA* and *NOX4*, and increases the levels of *SOD* and hydrogen peroxide, which may occur by targeting *Semaphorin 3A* (*Sema3A*) (126). miR-124a expression in bone marrow stromal stem cells (BMSCs) has a protective effect on OS-induced podocyte damage; transfection of BMSCs with miR-124a inhibits the phosphoinositide 3-kinase/mTOR signaling pathway, thus protecting podocytes (127). This suggests that the combined effects of BMSCs and miRNA may be beneficial for the treatment of DN.

### lncRNAs and Diabetic Nephropathy-Related OS

lncRNA refers to a long-chain RNA molecule with a length >200 nt and no protein coding ability, and regulate the process of DN. The expression of the lncRNA KCNQ1OT1 is increased in the serum of patients with DN and in HG-treated HK-2 cells (125). Further, KCNQ1OT1 directly targets miR-506-3p, and therefore, the interference of KCNQ1OT1 expression promotes miR-506-3p expression, thereby inhibiting inflammation, OS, and pyroptosis in HG-treated HK-2 cells (125). Another study in HK-2 cells showed that the expression of the lncRNA GAS5 decreases in HG-treated HK-2 cells, while the overexpression of GAS5 reduces ROS and MDA levels, and increases SOD levels (108). miRNA-452-5p expression is increased in HG-treated HK-2 cells, and interference of GAS5 expression may reverse the effects of miRNA-452-5p on HG-induced inflammation, OS, and pyroptosis of renal tubular cells (108). Furthermore, lncRNA Blnc1 is highly expressed in the serum of patients with DN, STZ-induced DN models, and HG-treated HK-2 cells; it is involved in the occurrence and development of DN, and its interference significantly reduces renal fibrosis, inflammation, and OS (128). Wang et al. (129) found that Linc00462 is significantly upregulated in the kidneys of patients with DN, and that its level increases in a glucose concentration- and time-dependent manner in HG-treated HK-2 and HMC cells. Knockdown of Linc00462 significantly reduces the cell viability of HG-treated cells and the levels of ROS and MDA induced by HG, while increasing the levels of SOD and catalase. Therefore, it upregulates the antioxidant system against ROS, indicating that knocking out Linc00462 may be a potential treatment for DN.

In addition to renal tubular cells, impairment of lncRNA expression in glomerular cells also participates in DN-related OS. Zhang et al. (112) found that the expression of lncRNA CASC2 decreases in the serum of patients with DN and in HG-treated HMCs, while the upregulation of CASC2 inhibits HMC proliferation, ECM accumulation, and HG-induced OS. miR-133b, the target of CASC2, is highly expressed in the serum of patients with DN and in HG-treated HMCs; the enrichment in miR-133b reverses the effect of CASC2 upregulation. The study confirmed that the upregulation of CASC2 inhibits HMC cell proliferation, ECM accumulation, and HG-induced OS through the miR-133b/FOXP1 axis, suggesting that CASC2 may be used as a novel target for DN treatment (112). Linc01619 is downregulated in renal tissues of patients with DN, and is associated with proteinuria and decreased renal function (113). Further, *in vitro* experiments confirmed that Linc01619 is expressed in the cytoplasm of podocytes, participating in the ERS signaling pathway, where it may be used as a competitive endogenous RNA that regulates the ERS and podocyte damage mediated by miR-27a/FOXO1 in DN (113). These results indicate that lncRNAs play an important role in DN-related OS and may improve DN by affecting miRNA expression.

### Other ncRNAs and Diabetic Nephropathy-Related OS

circRNA is a large ncRNA, which binds to miRNA and terminates the regulation of its target genes, namely, the circRNA-miRNA-mRNA regulatory network. circLRP6 regulates HG-induced MC proliferation, OS, ECM accumulation, and inflammation by competitively binding to miR-205 and *HMGB1*, and activating the Toll-like receptor 4/NF-κB pathway (130). These findings provide a better understanding of the pathogenesis of DN. In addition, antisense mitochondrial non-coding RNA-2 (ASncmtRNA-2) is expressed in experimental DN models and *in vitro* in human renal mesangial cells (HRMCs). Furthermore, it is significantly upregulated in mice with hereditary type 2 DM that also develop DN. When using the nitric oxide synthase inhibitor NG-nitro-L-arginine methyl ester (L-NAME) to inhibit ROS, the upregulation of ASncmtRNA-2 in DN is significantly reduced. In cultured HRMCs, HG treatment upregulates ASncmtRNA-2 expression in a time-dependent manner. Incubation of HRMCs with L-NAME also reduces the glucose-induced upregulation of ASncmtRNA-2. In addition, ROS upregulate ASncmtRNA-2 and may promote glomerular fibrosis in DN by actively regulating the expression of pro-fibrotic factors (131). This indicates that ASncmtRNA-2 is involved in the DN-related OS response and renal fibrosis development.

## Potential Clinical Applications of Os-Related ncRNAs in DN

The morbidity and mortality of DN remains high worldwide. Many studies have shown that early and timely intervention in DN significantly limits proteinuria, thereby preventing further development of DN (132). Therefore, the identification of novel molecular markers and drug targets for DN prevention and treatment is urgently needed. The application of next-generation sequencing technologies for RNAseq revealed that changes in the expression of miRNAs are very common in the pathogenesis of diabetes and DN (133). In addition, miRNAs are widely present in various parts of the cell, increasing their potential as DN-specific molecular markers.

The expression of many miRNAs related to OS is different in diabetic and healthy people (134, 135). For example, OS-related miR-21 has been proposed as a diagnostic marker for prediabetes (135), and receiver operating characteristic (ROC) curve analysis has shown that the expression of miR-21 is a suitable candidate marker for distinguishing diabetes from prediabetes (sensitivity, 93%; specificity, 35%). The ROC area under the curve (AUC) was 0.7 (Table 1). Moreover, the serum levels of miR-99b, miR-486-5p, miR-122-5p, and miR-20a in the serum, whose target genes are closely related to OS (114), are considered to have diagnostic value in diabetic kidney diseases (Table 1).

**Table 1:** Diagnostic index of miRNA in human DM-related OS.

**miRNAs**	**Diseases**	**Sample numbers (control/diseases)**	**AUC**	**Sensitivity**	**Specificity**	OR (95% CI*)	**Ref**
miR-21	Diabetes	44/27	0.7	93%	35%	1.05 (1.01–1.09)	(121)
miR-99b	DKD	25/42	0.895	-	-	-	(114)
miR-486-5p	DKD	25/42	0.853	-	-	-	(114)
miR-122-5p	DKD	25/42	0.8	-	-	-	(114)
miR-20a	DKD	25/42	0.697	-	-	-	(114)

*DM, diabetes mellitus; AUC, area under the curve; OR, odds ratio; CI, confidence interval; Ref, reference; DKD, diabetic kidney disease*.

Although, ncRNAs are closely associated with DN-related OS, there are limited related clinical drug studies. Shao et al. (105) treated DN rats with ginsenoside Rb1, triclosan, and ginsenoside Rb1 plus trigonelline and found that the combination of ginsenoside Rb1 and trigonelline significantly alleviates renal dysfunction, OS, and pathological changes. Further, studies have confirmed that ginsenoside Rb1 and trigonelline regulate the expression of miR-3550, which regulates the Wnt/β-catenin signaling pathway, therefore preventing the occurrence of diabetic kidney disease (105). Sitagliptin, a dipeptidyl peptidase 4 inhibitor used for the treatment of type 2 DM, has a protective effect on diabetic chronic kidney diseases. Civantos et al. (136) conducted proteomic and miRNA transcriptomic analysis of the renal cortex of wild-type (Wistar), diabetic Goto-Kakizaki (GK) rats, and rats treated with siglitine. Proteomic analysis of diabetic GK and Wistar rats showed differential expression of 39 proteins, and significant changes occurred among 15 miRNAs in GK rats, which are mainly related to OS and catabolism. Further studies have confirmed that treatment with sitagliptin improves OS in experimental DN *via* the mir-200a/*Keap-1*/*Nrf2* antioxidant pathway, thus, exerting renal-protective effects. Ochratoxin A, a mycotoxin with nephrotoxic and potentially carcinogenic activity, induces the expression of miR-200c and miR-132 in renal proximal epithelial cells. miR-200c and miR-132 target *NRF2* and *HO-1*, respectively, thereby promoting renal OS and inducing renal injury (137). Thus, ncRNAs are considered potential therapeutic targets based on their regulatory roles. Further, research is necessary to explore the diagnostic value of OS-related ncRNAs in DN, to identify novel drug targets, and prevent DN.

## Conclusions

Recently, ncRNAs have been recognized as a “new star” in the field of DN. As a class of novel regulatory molecules, they participate in multiple steps of DN by modulating the expression of several related genes. The development of RNAseq and next generation sequencing technologies has allowed the identification of a large number of ncRNAs. The extraction and detection of ncRNAs has higher specificity and sensitivity than those of proteins. Thus, ncRNAs can potentially be used as potential diagnostic and prognostic biomarkers. In addition, we can silence or activate ncRNAs in DN patients by exogenous means. For example, we can wrap the silenced or active lentiviral vector of ncRNAs into exosomes or other vehicles *in vitro*, and perform a targeted injection into the corresponding organs *via* blood. This can be developed as a therapy for DN. Considering their roles in the progression of DN-related OS, ncRNAs have great potential as “biological tools” for the screening, diagnosis, and treatment of DN, and may ultimately cure DN. However, there is a long road from the scientific research of ncRNAs to their clinical application. Recently, the research on ncRNA and DN has faced a series of challenges and limitations: (1) many ncRNAs are yet to be discovered and identified in DN through the development of RNAseq and next generation sequencing technologies; (2) ncRNAs need to be further investigated to determine whether they are specifically related to one or more diseases, and to explore the underlying molecular mechanisms whereby ncRNAs contribute to the disease; (3) The exact and specific mechanisms whereby ncRNAs regulate DN-related OS remain to be discovered; (4) According to many animal models, ncRNAs play a role in DM and its complications; however, the lack of clinical trials confirming the accuracy and safety of these findings remains an issue; (5) Many endogenous and exogenous factors involved in ncRNA production have not yet been identified, which hinders the use of ncRNAs as clinical therapeutic targets for DN treatment.

In this review, the regulatory role of ncRNAs in DN-related OS has been summarized. These ncRNAs regulate individual target genes or constitute interaction networks, such as lncRNA-miRNA-mRNA or circRNA-miRNA-mRNA, and play an important role in the regulation of DN-related OS. As our understanding of the molecular mechanisms involved in ncRNA regulation and their function *in vitro* and *in vivo* increases, novel and more effective treatment methods will be developed, which may cure DN by targeting the corresponding key ncRNAs.

## Author Contributions

XH, GK, and YZ conceived the concept and wrote the manuscript. CO edited and improved the manuscript. All authors contributed to the article and approved the submitted version.

## Conflict of Interest

The authors declare that the research was conducted in the absence of any commercial or financial relationships that could be construed as a potential conflict of interest.
